# *Pistacia atlantica's* effect on ovary damage and oxidative stress in streptozotocin-induced diabetic rats

**DOI:** 10.5935/1518-0557.20200044

**Published:** 2021

**Authors:** Mohammad Amin Behmanesh, Seyedeh Mahsa Poormoosavi, Yousef pareidar, Behnam ghorbanzadeh, Ahmad Mahmoodi-kouhi, Hossein Najafzadehvarzi

**Affiliations:** 1 Department of Histology, School of Medicine, Dezful University of Medical Sciences, Dezful, Iran; 2 Department of Histology, School of Medicine, Research and Clinical Center for Infertility Dezful University of Medical Sciences, Dezful, Iran; 3 Department of Gastroenterology, School of Medicine, Dezful University of Medical Sciences, Dezful, Iran; 4 Department of pharmacology, School of Medicine, Dezful University of Medical Sciences, Dezful, Iran; 5 Student Research Committee, Dezful University of Medical Sciences, Dezful, Iran; 6 Cellular and Molecular Biology Research Center, Health Research Institute, Babol University of Medical Sciences, Babol, Iran

**Keywords:** *Pistacia atlantica* extract, diabetes, ovary, rat

## Abstract

**Objective:**

Diabetes mellitus (DM) is associated with numerous complications, including gonadal dysfunction. There are specific traditional medicine remedies for DM, including medicinal herbs. Our study aimed to evaluate the role of *Pistacia atlantica’s* extract in the protection against ovary damage by streptozotocin (STZ)-induced DM in rats.

**Methods:**

We ran this experimental study on 40 adult female Wistar rats. We divided the animals into five groups, control (A); DM (STZ by 60 mg/kg- intraperitoneally) (B); DM + hexane extract of *P. atlantica* (200 mg/kg -orally) (C); *P. atlantica* extract (D) and DM + glibenclamide (200 mg/kg -orally) (F). The experiment continued for four weeks, and we administered the extract daily. After slaughtering the rats, we removed the ovaries. We assessed parameters, such as blood glucose and levels of oxidative stress markers as well as histological ovary structure.

**Results:**

Blood glucose, malondialdehyde (MDA) levels, and the number of atretic follicles were elevated; catalase (CAT), superoxide dismutase (SOD) levels and the number of corpora lutea were significantly decreased in the untreated diabetic rats. These changes returned to normal or diminished with *P. atlantica* extract and glibenclamide in the treated rats.

**Conclusions:**

The extract of *P. atlantica* has antihyperglycemic and antioxidative properties, and it decreased ovarian complications in experimental diabetes mellitus.

## INTRODUCTION

Diabetes mellitus (DM) is a pathological condition, which is associated with numerous complications, such as metabolic disorders and tissue alterations. The etiology of DM involves oxidative stress ^(Coskun et al., 2005)^, which stems from poor antioxidant defense mechanisms and the production of reactive oxygen species (ROS) ^(Rajasekaran *et al*., 2005)^. In addition, DM causes some disorders in the function and structure of ovaries such as changes in the estrous cycle, decreasing number of luteal cell, disruption of ovarian steroidogenesis and follicle growth and ovulation ^(Garris *et al*., 1985)^.

Antioxidant defense mechanisms are physiological protective processes against free radical production and its subsequent complications ^(Halliwell & Gutteridge, 1984)^. These mechanisms change in patients with DM, which in turn diminishes the scavenging effects against free radicals to examine the level of tissue damage ^(Wohaieb & Godin, 1987)^.

In the studies on animal models with DM, such as the diabetic rats induced by alloxan or streptozotocin (STZ), high levels of oxidative stress have been reported, which could be caused by chronic hyperglycemia. Such conditions lead to the depletion of antioxidant defense, thereby increasing the risk of free radical production ^(Coskun et al., 2005^; ^Rajasekaran *et al*., 2005)^. In addition to diabetes type I, STZ causes oxidative stress, hyperglycemia, and subsequent complexities in pathology and etiology.

Insulin is released from pancreatic ß-cells through stimulation by sulfonylureas (e.g., glibenclamide), which are among the agents commonly used in the treatment of type I diabetes. Glibenclamide is also the drug of choice for the treatment of the models with moderate STZ-induced DM ^(Ivorra et al., 1988)^. However, sulfonylureas have limited application due to the high rate of secondary failure, various side effects, and pharmacokinetic features ^(Kameswara Rao et al., 1997)^. Consequently, researchers have been concerned with the discovery of new medicines for the treatment of DM. Some herbal medicines were evaluated for their anti-diabetic effects in experimental or clinical studies. Moreover, these plants have fewer toxic and side effects when compared to synthetic drugs ^(Pari & Umamaheswari, 2000)^. Medicinal herbs are abundant sources of antioxidants, such as vitamin C and E, tannins, flavonoids, and carotenoids. Another prominent feature of plant-based drugs is the ability in scavenging and preventing oxidative stress ^(Rajasekaran *et al*., 2005)^.

Alternative treatments for DM involve the use of medicinal herbs; such example is *Pistacia* plants known by the English common name Mt. Atlas mastic tree and the local name in Iran is Baneh, which belongs to the Anacardiaceae family and grow in the Zagross region (Iran) ^(Hashemnia et al., 2015)^. The plant epidermis contains variable levels of phenolic substances, which significantly lower the oxidation process pace ^(Saeb *et al*., 2005)^. In addition, the *Pistacia* herbal extract has antioxidant, antimicrobial, antiatherogenic, anti-inflammatory, hypoglycemic, and anti-insect effects ^(Hamdan & Afifi, 2004)^. In addition, it has diuretic and stimulant effects, as well as urinary and respiratory antiseptic properties ^(Ghalem & Mohamed, 2009)^. ^Eftekharafzali et al. (2018)^ reported tha *P. atlantica* is significantly effective without severe side effects in dyspepsia, in a clinical trial. *Pistacia atlantica* is used for multiple purposes, such as in some stomach diseases, renal disorders, wounds and coughs ^(Mahjoub et al., 2018)^.

The present study aimed to investigate the effects of *P. atlantica* herbal extract via oral administration on blood glucose levels and oxidative/antioxidant status and histomorphological changes in the ovaries of the rats with STZ-induced DM.

## MATERIALS AND METHODS

### Animal Housing and Animal Grouping

For this experimental study, we obtained forty adult female Wistar rats (10-14 weeks old, 200±20g) from the Dezful University of Medical Sciences Animal house (Dezful, Iran) and used them in a completely randomized design. We randomly distributed the animals, which had the same strain, color and age into groups. During the study, all the rats were kept under controlled light and dark condition (12-hour light/12-hour dark cycles) at room temperature (25±2ºC), and had free access to food and water. The Ethics Committee of Dezful University approved the study, and all the experiments were performed in accordance with the guidelines for the safe handling of animals ^(Smith *et al*., 2018)^.

We randomly divided the animals into five equal groups (n=8/group): Group (A) served as the control group and received a daily oral gavage of normal saline; Group (B) diabetic rats, received streptozotocin (STZ). Group (C), diabetic rats received *P. atlantica* extract (200 mg/kg) ^(Hashemnia et al., 2015)^ daily by gavage. Group (D) non-diabetic rats received similar doses of *P. atlantica* extract alone. Group (E) diabetic rats received glibenclamide (200 mg/kg) daily by gavage. All treatments were given daily by an oral gavage, for 4 weeks ^(Hosseinifar et al., 2011^; ^Behmanesh et al., 2018)^. At the end of the experiment, the rats were anaesthetized with sodium thiopental (30 mg/kg) and slaughtered. Then the ovaries were harvested.

We induced the diabetes in the rats through a single intraperitoneal injection of 60 mg/kg ^(Hosseinifar et al., 2011)^ of STZ (CAS No. 18883-66-4) Solarbio Company (China). Then, we fasted the rats for 12h before the STZ injection ^(ABD El-Kader et al., 2019)^. We selected those rats with blood glucose levels higher than 250 mg/dl, and with polydipsia and polyuria, for at least 3 days. During the course of the experiment, we measured their blood glucose once every two weeks (The first day of diabetes, 15 days after diabetes and 30 days after diabetes) ^(Behmanesh et al., 2017)^. We collected the blood from the tail of the animals after 12h fasting ^(James et al., 2016)^, using an Easy On-Call Extra Gluco glucometer (ACON, USA).

### *P. atlantica* preparation

We obtained the *P. atlantica* fruits from the local market and dried them using the bin drying method. We separated the *P. atlantica* seeds manually, from the pulp of the dried fruits and grounded it into powder. We extracted the powder using N-hexane (1:4, w/v) for 48 h in the dark. We pooled and concentrated the filtrates under vacuum at a temperature, not exceeding 60ºC. We stored the extracts at −20ºC until time to use it. We selected this protocol according to the ^Hashemnia et al. (2015)^ study.

### Biochemical and histological evaluation

We homogenized the weighed amounts of ovary tissues (20mg) ^(Ugochukwu *et al*., 2003)^, and the supernatant of homogenized ovary tissues was used for the activity of:

- superoxide dismutase (SOD), assayed by the ^Misra & Fridovich (1972)^ method.
- Catalase (CAT) was assayed by the ^Takahara *et al*. (1960)^ method.
- lipid peroxidation using malondialdehyde (MDA) levels was assayed by the ^Sunderman *et al*. (1985)^ method.

We fixed the ovary samples from each rat in 10% formalin. We prepared the 5-6µ sections, with 20µ intervals, using the paraffin embedding techniques with a rotary microtome (RM2235, Leica Company from USA). We used hematoxylin-eosin to stain the ovary for structural and histopathological examination. For the histomorphometric study, we used photomicrographs taken from sections using an Olympus optical microscope equipped with a dino lit camera, at a magnification of 4×, 10× and 40× and used the dino lit software to extract the data. We counted the numbers of primordial, primary and secondary follicles under a 40× magnification; we counted the antral and artesian follicle under a 10× microscopic magnification. In addition, we measured the diameter of follicles containing oocytes with germinal vesicles in each animal, three sections from one animal in each group, and three randomly chosen areas in each section. The sections were quantitatively (morphometric) and qualitatively (morphologic) evaluated. Base on the literatures and related research; ovarian dysfunction in diabetic rats is associated with decreasing number of follicles and follicular atrophy as well as changes in lipid peroxidation and antioxidant defense ^(Nayki et al., 2017^; ^Erbas et al., 2014)^.

### Statistical Analysis

We ran all the analyses using the SPSS version 16. We analyzed the group’s variance using the one-way Analysis of variance (ANOVA), and the Fisher’s least significant difference test (LSD) to evaluate significant differences between the groups. A *p*<0.05 was considered statistically significant.

## RESULTS

Table 1 depicts the effects of oral administration of the *P. atlantica* extracts and glibenclamide on the blood glucose in the diabetic rats. Blood glucose concentrations in the untreated diabetic rats were significantly higher than those of the other groups (*p*<0.05). There were no significant differences in blood glucose between treated diabetic groups (*p*<0.05). The control group did not show any significant alterations in blood glucose level during the course of the study.

**Table 1:** Mean ± SD of the blood glucose levels of rats in different groups

Groups	Parameter
	Blood glucose (mg/dl)
Group A	111.3±3.1^a^
Group B	379.1±4.2^a,b,c,d^
Group C	132.7±2.4^b^
Group D	110.2±2.3^c^
Group E	124.5±3.3^d^

Same superscript in each column indicate significant differences at *p*<0.05. Group (A) which served as the control group and received a daily oral gavage normal saline Group (B) diabetic rats, which received streptozotocin. Group (C) diabetic rats were administered *P. atlantica* extract (200 mg/kg) daily by gavage. Group (D) non-diabetic rats received similar doses of *P. atlantica* extract alone. Group (E) diabetic rats were administered glibenclamide (200 mg/kg) daily by gavage.

Table 2 shows the biomarkers associated with the antioxidative status in the various groups. In the course of diabetes, there was a significant (*p*<0.05) reduction in SOD and CAT activities. Treatment of diabetic rats with *P. atlantica* extract and glibenclamide increased SOD and CAT activities. There was no difference (*p*<0.05) in SOD and CAT levels among normal rats. There was a significantly (*p*<0.05) elevated level of MDA in diabetic control rats, compared with diabetic rats treated with *P. atlantica* and glibenclamide. There was no difference (*p*<0.05) in the levels of MDA among normal rats.

**Table 2:** Mean ± SD of the malondialdehyde (MDA), Catalase (CAT) and superoxide dismutase (SOD) levels of rats in different groups

Groups	Parameter		
	MDA (nmol/ml^-1)^	CAT (U/ml^-1)^	SOD (U/ml^-1)^
Group A	3.74±2.1^a^	9.45±0.9	8.27±0.5^a,b,c^
Group B	8.52±1.4^a,b,c,d^	3.34±0.7^a,b^	2.2±2.3^a,d^
Group C	3.32±0.9^b^	5.65±1.2^a^	4.68±1.3^b,e^
Group D	4.05±1.1^c^	9.5±1.1	7.88±0.6^d,e,f^
Group E	4.44±0.7^d^	5.11±2.2^b^	4.56±0.8^c,f^

Same superscript in each column indicate significant differences at *p*<0.05. Group (A) which served as the control group and received a daily oral gavage normal saline Group (B) diabetic rats, which received streptozotocin. Group (C) diabetic rats were administered *P. atlantica* extract (200 mg/kg) daily by gavage. Group (D) non-diabetic rats received similar doses of *P. atlantica* extract alone. Group (E) diabetic rats were administered glibenclamide (200 mg/kg) daily by gavage.

**Figure 1:**
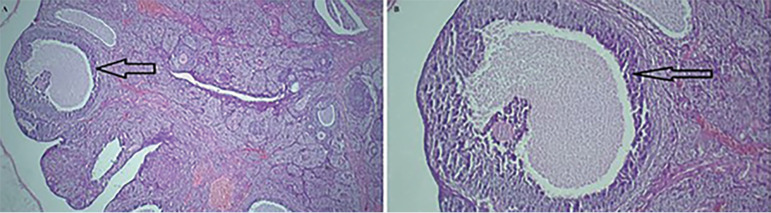
Light photomicrographs of an ovary in the control group. A. The structure of the ovarian tissue is normal with healthy growing follicles (H&E, ×40) and B. (H&E, ×100). Arrow: secondry follicle.

**Figure 2:**
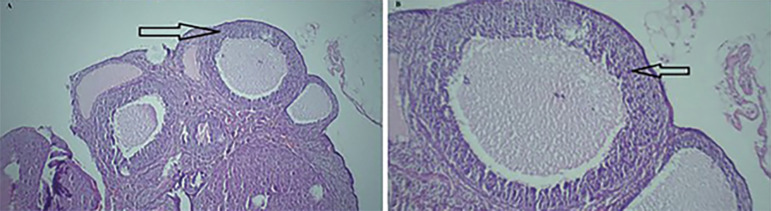
Light photomicrographs of an ovary in the diabetic group. A. Several atresian follicle (H&E, ×40) and B. (H&E, ×100). arrow: atresian follicle with dense and picnotic nuclei and segregation in granulosa cells.

**Figure 3:**
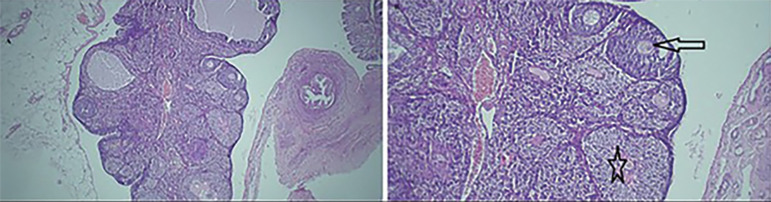
Light photomicrographs of the disease treatment group. A. The structure of the ovarian tissue is normal and there are various follicles along with corpus luteum (H&E, ×40) and B. (H&E, ×100). Arrow: secondary follicle. Star: corpus luteum.

Table 3 shows the number of corpora lutea, antral follicles, atretic follicles, and preovulatory follicles, of all groups. The mean number of atretic follicles increased while the mean number of secondary follicles and antral follicles reduced in untreated diabetic rats, when compared to the control group. Figure 1 (*p*≤0.05). In addition, untreated diabetic rats had a lower number of corpora lutea (Fig. 2) (*p*≤0.05). While the number of corpora lutea did not differ in normal and treated diabetic rats (Fig. 3). The mean total number of primordial and primary follicles showed no significant difference among the groups (*p*>0.05).

**Table 3:** Mean ± SD of the number of primordial, primary, secondary, antral, atretic follicles and corpus luteum of rats in different groups

Groups	Parameter					
	Primordial	Primary	Secondary	Antral	Atretic	Corpus luteum
Group A	1.35±2.4	2.24±1.6	4.45±0.7^a^	0.38±1.7^a^	1.45±0.5^a^	8.4±1.1^a^
Group B	1.12±2.0	2.11±1.4	2.61±2.0^a^	0.11±2.1^a^	2.87±0.7^a^	5.22±1.1^a,b,c,d^
Group C	1.14±1.1	2.12±1.2	4.32±2.1	0.25±0.9	1.90±1.2	7.71±2.1^b^
Group D	1.27±0.6	2.17±1.5	4.5±0.3	0.29±1.3	0.98±1.3	7.98±0.9^c^
Group E	1.20±1.5	2.16±0.5	4.37±2.1	0.30±0.7	1.39±1.4	8.05±0.7^d^

Same superscript in each column indicate significant differences at *p*<0.05. Group (A) which served as the control group and received a daily oral gavage normal saline Group (B) diabetic rats, which received streptozotocin. Group (C) diabetic rats were administered *P. atlantica* extract (200 mg/kg) daily by gavage. Group (D) non-diabetic rats received similar doses of *P. atlantica* extract alone. Group (E) diabetic rats were administered glibenclamide (200 mg/kg) daily by gavage.

Table 4 depicts the diameter (µ) of corpora lutea, antral follicles, atretic follicles, and preovulatory follicles, of all groups. The diameter of antral follicles and corpora lutea in untreated diabetic rats were decreased compared to the control group (*p*≤0.05). There were no significant differences between the diameter of the primordial, primary, secondary and atretic follicles in other groups (*p*>0.05). In diabetic rats treated with *P. atlantica*, all of the alterations were compensated to the Control’s level (*p*<0.05).

**Table 4:** Mean ± SD of the diameter (µ) of primordial, primary, secondary, antral, atretic follicles and corpus luteum of rats in different groups

Groups	Parameter					
	Primordial	Primary	Secondary	Antral	Atretic	Corpus luteum
Group A	32.2±0.4	42.1±0.5	209.1±2.1	1087.2±1.6^a^	921.0±1.1	995.3±0.9^a^
Group B	25.5±1.7	36.9±0.9	171±2.1	623.1±1.9^a^	897.1±1.5	557.9±1.5^a^
Group C	28.7±2.2	40.7±0.9	175±1.8	981.2±1.4	906.5±1.3	898±0.7
Group D	30.6±0.5	43.1±2.2	199±0.4	998.0±0.9	935.5±0.8	1025.4±1.1
Group E	27.9±0.9	37.1±0.7	201±0.9	995.9±1.2	911.0±1.7	1001.2±0.7

Same superscript in each column indicate significant differences at *p*<0.05. Group (A) which served as the control group and received a daily oral gavage normal saline Group (B) diabetic rats, which received streptozotocin. Group (C) diabetic rats were administered *P. atlantica* extract (200 mg/kg) daily by gavage. Group (D) non-diabetic rats received similar doses of *P. atlantica* extract alone. Group (E) diabetic rats were administered glibenclamide (200 mg/kg) daily by gavage.

## DISCUSSION

According to the literature, *P. atlantica* has antioxidant, antimicrobial, antiatherogenic, anti-inflammatory, hypoglycemic, and anti-insect properties ^(Giner-Larza et al., 2000^; ^Dedoussis et al., 2004)^. However, no studies have investigated the effects of *P. atlantica* on improving ovarian function in the rats with DM. No serious side effects have been reported for this plant, but like other herbal plants, it can cause complications and poisoning if not properly identified or improperly prepared, or even used correctly.

According to the findings of the current study, the level of blood glucose was higher in the diabetic rats with no treatment than in healthy animals. STZ induced an experimental model of diabetes type I along with several complications, including the effects of oxidative stress of the reproductive system ^(Eddouks et al., 2004)^. Furthermore, *P. atlantica* herbal extract could significantly reduce blood glucose levels in rats with STZ-induced DM, which could be attributed to the inhibitory *in-vitro* effects of α-glucosidase and α-amylase ^(Hamdan & Afifi, 2004^; ^Kasabri et al., 2011)^. These findings are in congruence with the study by ^Hashemnia et al. (2015)^.

The mechanism of *P. atlantica* in decreasing hyperglycemia remains unclear, although previous findings in these regards suggested mechanisms such as insulin-like function, facilitation of insulin activity, inhibition of insulinase activity, interference of the fiber content of plants with carbohydrate absorption, inhibiting the production of hepatic glucose and stimulating glucose use by the peripheral tissues ^(Giner-Larza et al., 2000^; ^Oryan et al., 2014)^.

Contrary to these findings, ^Hamdan & Afifi (2004)^ rejected the hypoglycemic effects of *P. atlantica*. In the present study, oral administration of *P. atlantica* to diabetic rats exerted hypoglycemic effects, thereby enhancing oxidative stress. Additionally, the herbal treatment was observed to significantly increase the MDA concentration in the body tissues of the diabetic rats, while decreasing the antioxidant effects of SOD and CAT. This is in line with the study by ^Farhoosh et al. (2009)^. According to the aforementioned studies, using the leaves *P. atlantica* exerted similar or more significant antioxidant effects when compared to conventional antioxidant agents, *in-vitro* ^(Bahmani et al., 2015)^.

The kernel oil found in *P. atlantica* has remarkable antioxidant properties ^(Nayki et al., 2017)^. Histological evaluations in the current research indicated increased atretic follicles and reduced antral follicles, while the diabetic animals had corpus luteum and secondary follicles as well. Moreover, there was induced apoptosis in the granulosa cells, decreasing the diameter of corpora lutea and antral follicles in diabetic rats ^(Tatewaki *et al*., 1989)^.

Rats with DM demonstrated ovarian dysfunction due to imbalanced glucose deployment, impaired steroidogenesis, and follicular atrophy. In our study, treatment with *P. atlantica* resulted in the compensation of these changes in diabetic rats, when compared to the control group, which could be attributed to increased antioxidant activity and decreased lipid peroxidation ^(Nayki et al., 2017)^. Moreover, the compounds found in *P. atlantica* allow remarkable significant protective antioxidant effects on the ovaries of diabetic rats. The hypothesis has been examined in several other studies using medicinal herbs with known antioxidant properties. According to the current research, treatment of DM with *P. atlantica* was associated with the effective inhibition of lipid peroxidation, as well as increased antioxidant capacity in the rats with STZ-induced diabetes.

Limitation of present study: induction diabetes mellitus in rats and access to biochemical kits limited our study.

## CONCLUSION

According to our results, *P. atlantica* could be used in the treatment of rats with STZ-induced diabetes, in order to diminish ovarian complications. It is notable that oxidative stress pathways may also be involved in the diabetic complications affecting the ovaries. *P. atlantica* reduced the oxidative stress through improving the antioxidant activities in rat ovaries. However, we recommend further experimental and clinical research on infertile women before using this herbal treatment as a routine procedure.

## References

[r1] ABD El-Kader AM, Awadalla AE, Gabr SA, Nour AH (2019). Evaluation of the histopathological and biochemical effect of Aloe Vera aqueous extract on diabetes mellitus induced by streptozotocin in rats. Asian J Pharm Clin Res..

[r2] Bahmani M, Saki K, Asadbeygi M, Adineh A, Saberianpour S, Rafieian-Kopaei M, Bahmani F, Bahmani E (2015). The effects of nutritional and medicinal mastic herb (Pistacia atlantica). J Chem Pharmaceut Res..

[r3] Behmanesh MA, Efani Majd N, Shahriari A, Najafzadeh H (2017). Evaluation of antioxidant potential of Aloe vera and pituitary sexual hormones after experimental diabetes in male rats. Iran J Vet Med..

[r4] Behmanesh MA, Najafzadehvarzi H, Poormoosavi SM (2018). Protective Effect of Aloe vera Extract against Bisphenol A Induced Testicular Toxicity in Wistar Rats. Cell J..

[r5] Coskun O, Kanter M, Korkmaz A, Oter S (2005). Quercetin, a flavonoid antioxidant, prevents and protects streptozotocin-induced oxidative stress and beta-cell damage in rat pancreas. Pharmacol Res..

[r6] Dedoussis GV, Kaliora AC, Psarras S, Chiou A, Mylona A, Papadopoulos NG, Andrikopoulos NK (2004). Antiatherogenic effect of Pistacia lentiscus via GSH restoration and downregulation of CD36 mRNA expression. Atherosclerosis.

[r7] Eddouks M, Lemhadri A, Michel JB (2004). Caraway and caper: potential anti-hyperglycaemic plants in diabetic rats. J Ethnopharmacol.

[r8] Eftekharafzali M, Mehrabani M, Tajadini H, Ahmadi B, Zahedi MJ (2018). Effect of “Pistacia Atlantica” Resin (Baneh) on Functional Dyspepsia: A Double Blind, Randomized Clinical Study. Iran Red Crescent Med J.

[r9] Erbas O, Pala HG, Pala EE, Oltulu F, Aktug H, Yavasoglu A, Taskiran D (2014). Ovarian failure in diabetic rat model: nuclear factor-kappaB, oxidative stress, and pentraxin-3. Taiwan J Obstet Gynecol.

[r10] Farhoosh R, Khodaparast MHH, Sharif A (2009). Bene hull oil as a highly stable and antioxidative vegetable oil. Eur J Lipid Sci Technol.

[r11] Garris DR, Williams SK, West L (1985). Morphometric evaluation of diabetes‐associated ovarian atrophy in the C57BL/KsJ mouse: relationship to age and ovarian function. Anat Rec..

[r12] Ghalem BR, Mohamed B (2009). Essential oil from gum of Pistacia atlantica Desf.: screening of antimicrobial activity. Afr J Pharm Pharmacol..

[r13] Giner-Larza EM, Máñez S, Giner-Pons RM, Carmen Recio M, Ríos JL (2000). On the anti-inflammatory and anti-phospholipase A(2) activity of extracts from lanostane-rich species. J Ethnopharmacol.

[r14] Halliwell B, Gutteridge JM (1984). Lipid peroxidation, oxygen radicals, cell damage, and antioxidant therapy. Lancet.

[r15] Hamdan II, Afifi FU (2004). Studies on the in vitro and in vivo hypoglycemic activities of some medicinal plants used in treatment of diabetes in Jordanian traditional medicine. J Ethnopharmacol.

[r16] Hashemnia M, Nikousefat Z, Yazdani-Rostam M (2015). Antidiabetic effect of Pistacia atlantica and Amygdalus scoparia in streptozotocin-induced diabetic mice. Comp Clin Pathol.

[r17] Hosseinifar SH, Erfanimajd N, Morovvati H, Najafzadeh H (2011). Aloe vera gel protects ovarian structure in diabetic rat. Am Eurasian J Toxicol Sci.

[r18] Ivorra MD, Paya M, Villar A (1988). Hypoglycemic and insulin release effects of tormentic acid: a new hypoglycemic natural product. Planta Med.

[r19] James SA, Omwirhiren REM, Joshua IA, Dutse I (2016). Anti-diabetic properties and phytochemical studies of ethanolic leaf extracts of Murraya koenigii and Telfairia occidentalis on Alloxan-induced diabetic albino rats. Adv Life Sci Technol.

[r20] Kameswara Rao B, Giri R, Kesavulu M, Apparao C (1997). Herbal medicine in the management of diabetes mellitus. Manphar Vaidhya Patrika.

[r21] Kasabri V, Afifi FU, Hamdan I (2011). In vitro and in vivo acute antihyperglycemic effects of five selected indigenous plants from Jordan used in traditional medicine. J Ethnopharmacol.

[r22] Mahjoub F, Akhavan Rezayat K, Yousefi M, Mohebbi M, Salari R (2018). Pistacia atlantica Desf. A review of its traditional uses, phytochemicals and pharmacology. J Med Life.

[r23] Misra HP, Fridovich I (1972). The role of superoxide anion in the autoxidation of epinephrine and a simple assay for superoxide dismutase. J Biol Chem..

[r24] Nayki C, Nayki U, Kulhan M, Ozkaraca M, Altun S, Cankaya M, Onk OA, Ulug P (2017). The effect of diabetes on ovaries in a rat model: the role of interleukin-33 and apoptosis. Gynecol Endocrinol.

[r25] Oryan A, Hashemnia M, Hamidi AR, Mohammadalipour A (2014). Effects of hydro-ethanol extract of Citrullus colocynthis on blood glucose levels and pathology of organs in alloxan-induced diabetic rats. Asian Pac J Trop Dis.

[r26] Pari L, Umamaheswari J (2000). Antihyperglycaemic activity of Musa sapientum flowers: effect on lipid peroxidation in alloxan diabetic rats. Phytother Res.

[r27] Rajasekaran S, Sivagnanam K, Subramanian S (2005). Antioxidant effect of Aloe vera gel extract in streptozotocin-induced diabetes in rats. Pharmacol Rep.

[r28] Saeb M, Jalaei J, Nazifi S, Mirzaei A (2005). Studies on the effects of turpentine powder on the serum concentration of lipids and lipoproteins of female rabbits. J Vet Res.

[r29] Smith AJ, Clutton RE, Lilley E, Hansen KEA, Brattelid T (2018). PREPARE: guidelines for planning animal research and testing. Lab Anim.

[r30] Sunderman F, Marzouk A, Hopfer S, Zaharia O, Reid M (1985). Increased lipid peroxidation in tissues of nickel chloride- treated rats. Ann Clin Lab Sci.

[r31] Takahara S, Hamilton HB, Neel JV, Kobara TY, Ogura Y, Nishimura ET (1960). Hypocatalasemia: a new genetic carrier state. J Clin Invest.

[r32] Tatewaki R, Otani H, Tanaka O, Kitada J (1989). A morphological study on the reproductive organs as a possible cause of developmental abnormalities in diabetic NOD mice. Histol Histopathol..

[r33] Ugochukwu N, Babady N, Cobourne M, Gasset S (2003). The effect of Gongronema latifolium extracts on serum lipid profile and oxidative stress in hepatocytes of diabetic rats. J Biosci.

[r34] Wohaieb SA, Godin DV (1987). Alterations in free radical tissue-defense mechanisms in streptozocin-induced diabetes in rat: effects of insulin treatment.. Diabetes.

